# 3,5-Bis(4-methoxy­phen­yl)-1*H*-1,2,4-triazole monohydrate

**DOI:** 10.1107/S160053680901695X

**Published:** 2009-05-14

**Authors:** Hai-Ying Wang, Jian-Ping Ma, Ru-Qi Huang, Yu-Bin Dong

**Affiliations:** a College of Chemistry, Chemical Engineering and Materials Science, Shandong Normal University, Jinan 250014, People’s Republic of China

## Abstract

In the title compound, C_16_H_15_N_3_O_2_·H_2_O, the two benzene rings and the triazole ring lie almost in the same plane, the triazole ring forming dihedral angles of 5.07 (9) and 5.80 (8)° with the benzene rings. In the crystal, there are three relatively strong inter­molecular O—H⋯N and N—H⋯O hydrogen bonds, which lead to the formation of a one-dimensional double chain running parallel to the *a* axis. Weak π—π inter­actions between the benzene rings of neighboring chains with a centroid–centroid distance of 3.893 (4) Å result in the formation of layers parallel to the *ac* plane.

## Related literature

For the biological activity and pharmaceutical applications of compounds containing triazole subunits, see: Chai *et al.* (2009 ▶); Nadkarni *et al.* (2001 ▶); Zhan & Lou (2007 ▶). For triazole ring bond-length data, see; Claramunt *et al.* (2001 ▶); Zhou *et al.* (2001 ▶); John (1998 ▶).
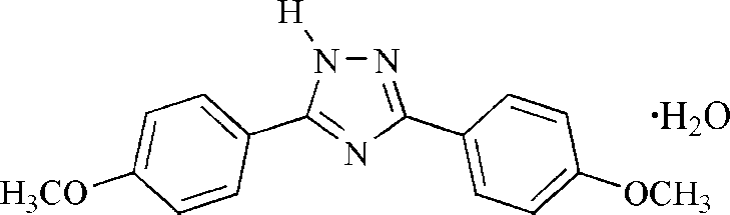


## Experimental

### 

#### Crystal data

C_16_H_15_N_3_O_2_·H_2_O
*M* *_r_* = 299.33
Triclinic, 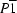

*a* = 6.9948 (18) Å
*b* = 11.125 (3) Å
*c* = 11.184 (3) Å
α = 110.603 (4)°
β = 107.932 (3)°
γ = 95.690 (4)°
*V* = 753.8 (3) Å^3^
*Z* = 2
Mo *K*α radiation
μ = 0.09 mm^−1^
*T* = 298 K
0.40 × 0.20 × 0.19 mm

#### Data collection

Bruker SMART CCD area-detector diffractometer
Absorption correction: none
3854 measured reflections
2651 independent reflections
1993 reflections with *I* > 2σ(*I*)
*R*_int_ = 0.016

#### Refinement

*R*[*F* ^2^ > 2σ(*F* ^2^)] = 0.052
*wR*(*F* ^2^) = 0.134
*S* = 1.05
2651 reflections
201 parameters
H-atom parameters constrained
Δρ_max_ = 0.17 e Å^−3^
Δρ_min_ = −0.25 e Å^−3^

### 

Data collection: *SMART* (Bruker, 2000 ▶); cell refinement: *SAINT* (Bruker, 2000 ▶); data reduction: *SAINT*; program(s) used to solve structure: *SHELXS97* (Sheldrick, 2008 ▶); program(s) used to refine structure: *SHELXL97* (Sheldrick, 2008 ▶); molecular graphics: *SHELXTL* (Sheldrick, 2008 ▶); software used to prepare material for publication: *SHELXTL*.

## Supplementary Material

Crystal structure: contains datablocks I, global. DOI: 10.1107/S160053680901695X/zl2201sup1.cif

Structure factors: contains datablocks I. DOI: 10.1107/S160053680901695X/zl2201Isup2.hkl

Additional supplementary materials:  crystallographic information; 3D view; checkCIF report

## Figures and Tables

**Table 1:** Hydrogen-bond geometry (Å, °)

*D*—H⋯*A*	*D*—H	H⋯*A*	*D*⋯*A*	*D*—H⋯*A*
O3—H3*A*⋯N1	0.97	1.96	2.902 (2)	164
N2—H2⋯O3^i^	0.86	1.90	2.753 (2)	170
O3—H3*B*⋯N3^ii^	0.96	1.97	2.885 (2)	159

Symmetry codes: (i) 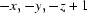; (ii) 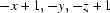.

**Fig. 1.**
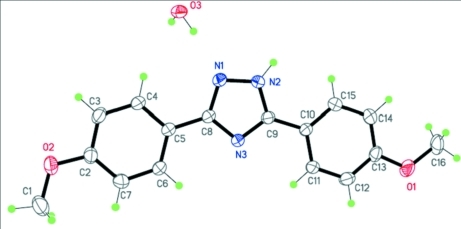
The molecular structure, with atom labels and 30% probability displacement ellipsoids.

**Fig. 2.**
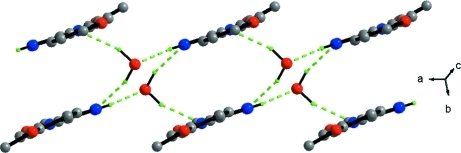
View of a one dimensional double chain of the title structure. Hydrogen bonds are shown as dashed lines. H atoms not involved in hydrogen bonding have been omitted for clarity.

**Fig. 3.**
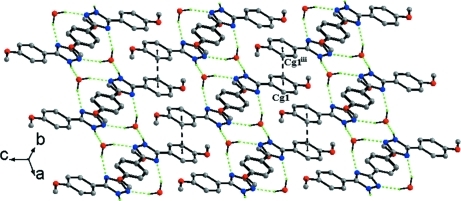
The crystal packing of the title compound via weak π—π interactions. The distance of centroids is 3.893 (4) Å (Dashed lines: hydrogen bonds; broken lines: π—π interactions.) [symmetry code: (iii) -x, -y, -z].

## supplementary crystallographic information

### Comment

During the past decades, compounds containing triazole subunits have been intensively studied due to their diverse biological activities, such as antibacterial, antitumor, *etc.* and have become a central focus in the study of agricultural and medicinal chemicals (Chai *et al.*, 2009; Nadkarni *et al.*, 2001; Zhan *et al.*, 2007). In a search for more effective antibacterial compounds, we have synthesized the title compound and determined its structure.

The molecular structure of the title compound is shown in Fig. 1. The two benzene rings and the triazole ring almost lie in the same plane. The corresponding dihedral angles of each benzene ring with the triazole ring are 5.07 (9) (between C2–C7 and N1–N3/C8/C9) and 5.80 (8)° (between N1–N3/C8/C9 and C10–C15), respectively. The bond lengths of the triazole ring are very similar to other 1*H*-1,2,4-triazole derivatives (Claramunt *et al.*, 2001; Zhou *et al.*, 2001). C8—N3 (1.365 (2) Å) and N1—N2 (1.359 (2) Å) are typical for carbon-nitrogen single bonds and nitrogen-nitrogen single bonds, and C8—N1 (1.323 (2) Å) and C9—N3 (1.330 (2) Å) correspond to typical carbon-nitrogen double bonds (John, 1998). C9—N2 (1.333 (2) Å) is a carbon-nitrogen single bond, but the bond length is markedly shorter than usual carbon-nitrogen single bonds and close to a double bond due to its conjugation with the C9—N3 double bond.

The packing of the molecules in the crystal structure is stabilized through N—H···O, O—H···O and π—π interactions. Water molecules act both as hydrogen-acceptor and as hydrogen-donor which leads to the formation of a one dimensional double chain running parallel to the *a* axis (Fig. 2, Table 1). The ring made up of C10 to C15 (with the centroid *Cg*1) is parallel to its symmetry related counterpart with a *Cg*1··· *Cg*1^iii^ distance of 3.893 (4) Å [symmetry code: (iii)-*x*, -*y*, -*z*]. Adjacent chains are linked *via* these intermolecular π—π interactions between the *Cg*1 rings to form a two-dimentional layer parallel to the *ac* plane (Fig. 3).

### Experimental

A mixture of 4-methoxyphenylmethylenemalononitrile (20 mmol), hydrazine dihydrochloride (20 mmol) and hydrazine hydrate (60 mmol) in ethylene glycol (10 ml) was heated to 403 K with stirring for 3–4 h. After cooling to room temperature, the reaction mixture was diluted with water (20 ml). The precipitate was filtered, washed with water, dried and purified by column chromatography on silica gel using CH_2_Cl_2_ as the eluent to afford a white solid after evaporation of the solvent. The white solid was dissolved in ethanol and colourless crystals of the title compound were obtained on slow evaporation of the solvent at room temperature.

### Refinement

Hydrogen atoms attached to carbon were placed in geometrically idealized positions (C_arene_—H = 0.93 Å, C_methyl_—H = 0.96 Å) and refined using a riding model with isotropic displacement parameters *U*_iso_ = 1.2 (1.5 for methyl groups) *U*_eq_(C). The H atoms attached to N and O atoms were located by Fourier difference synthesis and refined using a riding model with isotropic displacement parameters of *U*_iso_ = 1.2 *U*_eq_(N) and *U*_iso_ = 1.5 *U*_eq_(O).

### Crystal data

**Table:**

C_16_H_15_N_3_O_2_·H_2_O	*Z* = 2
*M**_r_* = 299.33	*F*(000) = 316
Triclinic, *P*1	*D*_x_ = 1.319 Mg m^−^^3^
Hall symbol: -P 1	Mo *K*α radiation, λ = 0.71073 Å
*a* = 6.9948 (18) Å	Cell parameters from 1120 reflections
*b* = 11.125 (3) Å	θ = 2.2–24.0°
*c* = 11.184 (3) Å	µ = 0.09 mm^−^^1^
α = 110.603 (4)°	*T* = 298 K
β = 107.932 (3)°	Block, colourless
γ = 95.690 (4)°	0.40 × 0.20 × 0.19 mm
*V* = 753.8 (3) Å^3^	

### Data collection

**Table:**

Bruker SMART CCD area-detector diffractometer	1993 reflections with *I* > 2σ(*I*)
Radiation source: fine-focus sealed tube	*R*_int_ = 0.016
graphite	θ_max_ = 25.1°, θ_min_ = 2.0°
phi and ω scans	*h* = −4→8
3854 measured reflections	*k* = −13→11
2651 independent reflections	*l* = −12→13

### Refinement

**Table:**

Refinement on *F*^2^	Primary atom site location: structure-invariant direct methods
Least-squares matrix: full	Secondary atom site location: difference Fourier map
*R*[*F*^2^ > 2σ(*F*^2^)] = 0.052	Hydrogen site location: inferred from neighbouring sites
*wR*(*F*^2^) = 0.134	H-atom parameters constrained
*S* = 1.05	*w* = 1/[σ^2^(*F*_o_^2^) + (0.0708*P*)^2^ + 0.0124*P*] where *P* = (*F*_o_^2^ + 2*F*_c_^2^)/3
2651 reflections	(Δ/σ)_max_ = 0.001
201 parameters	Δρ_max_ = 0.17 e Å^−^^3^
0 restraints	Δρ_min_ = −0.25 e Å^−^^3^

### Special details

**Table:**

Geometry. All e.s.d.'s (except the e.s.d. in the dihedral angle between two l.s. planes) are estimated using the full covariance matrix. The cell e.s.d.'s are taken into account individually in the estimation of e.s.d.'s in distances, angles and torsion angles; correlations between e.s.d.'s in cell parameters are only used when they are defined by crystal symmetry. An approximate (isotropic) treatment of cell e.s.d.'s is used for estimating e.s.d.'s involving l.s. planes.
Refinement. Refinement of *F*^2^ against ALL reflections. The weighted *R*-factor *wR* and goodness of fit *S* are based on *F*^2^, conventional *R*-factors *R* are based on *F*, with *F* set to zero for negative *F*^2^. The threshold expression of *F*^2^ > σ(*F*^2^) is used only for calculating *R*-factors(gt) *etc*. and is not relevant to the choice of reflections for refinement. *R*-factors based on *F*^2^ are statistically about twice as large as those based on *F*, and *R*- factors based on ALL data will be even larger.

### Fractional atomic coordinates and isotropic or equivalent isotropic displacement parameters (Å^2^)

**Table:**

	*x*	*y*	*z*	*U*_iso_*/*U*_eq_	
C1	1.3626 (4)	0.5268 (3)	0.8502 (3)	0.0831 (8)	
H1A	1.4171	0.4539	0.8091	0.125*	
H1B	1.4672	0.5887	0.9349	0.125*	
H1C	1.3199	0.5696	0.7886	0.125*	
C2	1.0219 (3)	0.3942 (2)	0.7668 (2)	0.0493 (5)	
C3	0.8500 (3)	0.3622 (2)	0.7965 (2)	0.0506 (5)	
H3	0.8550	0.3978	0.8864	0.061*	
C4	0.6729 (3)	0.27787 (19)	0.6928 (2)	0.0447 (5)	
H4	0.5585	0.2571	0.7137	0.054*	
C5	0.6599 (3)	0.22255 (18)	0.55736 (19)	0.0394 (5)	
C6	0.8338 (3)	0.2549 (2)	0.5307 (2)	0.0524 (6)	
H6	0.8292	0.2193	0.4409	0.063*	
C7	1.0141 (3)	0.3388 (2)	0.6341 (2)	0.0578 (6)	
H7	1.1300	0.3576	0.6138	0.069*	
C8	0.4703 (3)	0.13404 (18)	0.44671 (18)	0.0368 (4)	
C9	0.2616 (3)	0.00131 (18)	0.24816 (19)	0.0379 (5)	
C10	0.1682 (3)	−0.08505 (18)	0.10190 (19)	0.0393 (5)	
C11	0.2847 (3)	−0.0918 (2)	0.0197 (2)	0.0519 (6)	
H11	0.4199	−0.0417	0.0587	0.062*	
C12	0.2030 (3)	−0.1712 (2)	−0.1177 (2)	0.0598 (6)	
H12	0.2828	−0.1741	−0.1711	0.072*	
C13	0.0040 (3)	−0.2468 (2)	−0.1776 (2)	0.0503 (5)	
C14	−0.1137 (3)	−0.2420 (2)	−0.0979 (2)	0.0539 (6)	
H14	−0.2481	−0.2932	−0.1372	0.065*	
C15	−0.0313 (3)	−0.1610 (2)	0.0403 (2)	0.0499 (5)	
H15	−0.1121	−0.1576	0.0932	0.060*	
C16	−0.2664 (4)	−0.4015 (2)	−0.3827 (2)	0.0745 (8)	
H16A	−0.2851	−0.4629	−0.3425	0.112*	
H16B	−0.2915	−0.4491	−0.4783	0.112*	
H16C	−0.3617	−0.3455	−0.3735	0.112*	
N1	0.2943 (2)	0.10899 (16)	0.46420 (16)	0.0437 (4)	
N2	0.1636 (2)	0.02456 (16)	0.33612 (16)	0.0424 (4)	
H2	0.0354	−0.0092	0.3148	0.051*	
N3	0.4569 (2)	0.06990 (15)	0.31449 (15)	0.0404 (4)	
O1	−0.0613 (2)	−0.32325 (16)	−0.31464 (15)	0.0726 (5)	
O2	1.1911 (2)	0.48023 (16)	0.87639 (15)	0.0693 (5)	
O3	0.2326 (2)	0.08148 (15)	0.69915 (14)	0.0540 (4)	
H3A	0.2339	0.1008	0.6215	0.100*	
H3B	0.3314	0.0294	0.7142	0.100*	

### Atomic displacement parameters (Å^2^)

**Table:**

	*U*^11^	*U*^22^	*U*^33^	*U*^12^	*U*^13^	*U*^23^
C1	0.0486 (14)	0.087 (2)	0.079 (2)	−0.0039 (13)	0.0166 (13)	0.0062 (16)
C2	0.0446 (12)	0.0498 (13)	0.0414 (12)	0.0095 (10)	0.0117 (10)	0.0088 (10)
C3	0.0611 (14)	0.0521 (13)	0.0338 (12)	0.0116 (10)	0.0194 (10)	0.0106 (10)
C4	0.0481 (12)	0.0474 (12)	0.0392 (12)	0.0092 (9)	0.0215 (9)	0.0139 (10)
C5	0.0434 (11)	0.0407 (11)	0.0372 (11)	0.0122 (9)	0.0182 (9)	0.0157 (9)
C6	0.0494 (13)	0.0623 (14)	0.0362 (12)	0.0052 (10)	0.0205 (10)	0.0075 (10)
C7	0.0457 (12)	0.0662 (15)	0.0519 (14)	0.0043 (11)	0.0238 (11)	0.0103 (12)
C8	0.0401 (10)	0.0403 (11)	0.0340 (11)	0.0121 (8)	0.0173 (8)	0.0157 (9)
C9	0.0373 (10)	0.0439 (11)	0.0381 (11)	0.0114 (9)	0.0175 (9)	0.0191 (10)
C10	0.0417 (11)	0.0419 (11)	0.0351 (11)	0.0075 (9)	0.0147 (9)	0.0166 (9)
C11	0.0477 (12)	0.0585 (14)	0.0380 (12)	−0.0078 (10)	0.0166 (10)	0.0109 (11)
C12	0.0638 (15)	0.0636 (15)	0.0438 (13)	−0.0072 (12)	0.0258 (11)	0.0130 (12)
C13	0.0615 (14)	0.0427 (12)	0.0346 (12)	0.0000 (10)	0.0095 (10)	0.0123 (10)
C14	0.0437 (12)	0.0563 (14)	0.0495 (14)	−0.0016 (10)	0.0101 (10)	0.0170 (11)
C15	0.0397 (11)	0.0608 (14)	0.0475 (13)	0.0072 (10)	0.0173 (10)	0.0202 (11)
C16	0.0777 (17)	0.0606 (16)	0.0490 (15)	−0.0158 (13)	−0.0073 (12)	0.0160 (13)
N1	0.0420 (9)	0.0526 (10)	0.0366 (10)	0.0123 (8)	0.0178 (8)	0.0148 (8)
N2	0.0329 (8)	0.0539 (10)	0.0385 (10)	0.0071 (7)	0.0147 (7)	0.0160 (8)
N3	0.0370 (9)	0.0472 (10)	0.0338 (9)	0.0064 (7)	0.0148 (7)	0.0124 (8)
O1	0.0834 (12)	0.0678 (11)	0.0387 (10)	−0.0165 (9)	0.0129 (8)	0.0074 (8)
O2	0.0503 (9)	0.0764 (12)	0.0499 (10)	−0.0021 (8)	0.0085 (7)	0.0033 (9)
O3	0.0439 (8)	0.0746 (10)	0.0513 (9)	0.0170 (7)	0.0265 (7)	0.0255 (8)

### Geometric parameters (Å, °)

**Table:**

C1—O2	1.412 (3)	C9—C10	1.462 (3)
C1—H1A	0.9600	C10—C15	1.379 (3)
C1—H1B	0.9600	C10—C11	1.393 (3)
C1—H1C	0.9600	C11—C12	1.368 (3)
C2—O2	1.370 (2)	C11—H11	0.9300
C2—C7	1.373 (3)	C12—C13	1.375 (3)
C2—C3	1.389 (3)	C12—H12	0.9300
C3—C4	1.370 (3)	C13—O1	1.362 (2)
C3—H3	0.9300	C13—C14	1.380 (3)
C4—C5	1.390 (3)	C14—C15	1.379 (3)
C4—H4	0.9300	C14—H14	0.9300
C5—C6	1.381 (3)	C15—H15	0.9300
C5—C8	1.461 (3)	C16—O1	1.418 (3)
C6—C7	1.381 (3)	C16—H16A	0.9600
C6—H6	0.9300	C16—H16B	0.9600
C7—H7	0.9300	C16—H16C	0.9600
C8—N1	1.323 (2)	N1—N2	1.359 (2)
C8—N3	1.365 (2)	N2—H2	0.8600
C9—N3	1.330 (2)	O3—H3A	0.9678
C9—N2	1.333 (2)	O3—H3B	0.9583
			
O2—C1—H1A	109.5	C15—C10—C9	123.15 (18)
O2—C1—H1B	109.5	C11—C10—C9	119.01 (17)
H1A—C1—H1B	109.5	C12—C11—C10	120.85 (18)
O2—C1—H1C	109.5	C12—C11—H11	119.6
H1A—C1—H1C	109.5	C10—C11—H11	119.6
H1B—C1—H1C	109.5	C11—C12—C13	120.7 (2)
O2—C2—C7	124.66 (19)	C11—C12—H12	119.7
O2—C2—C3	115.73 (19)	C13—C12—H12	119.7
C7—C2—C3	119.61 (19)	O1—C13—C12	115.9 (2)
C4—C3—C2	119.73 (19)	O1—C13—C14	124.71 (19)
C4—C3—H3	120.1	C12—C13—C14	119.4 (2)
C2—C3—H3	120.1	C15—C14—C13	119.77 (19)
C3—C4—C5	121.72 (19)	C15—C14—H14	120.1
C3—C4—H4	119.1	C13—C14—H14	120.1
C5—C4—H4	119.1	C10—C15—C14	121.47 (19)
C6—C5—C4	117.40 (18)	C10—C15—H15	119.3
C6—C5—C8	120.93 (17)	C14—C15—H15	119.3
C4—C5—C8	121.67 (17)	O1—C16—H16A	109.5
C7—C6—C5	121.7 (2)	O1—C16—H16B	109.5
C7—C6—H6	119.2	H16A—C16—H16B	109.5
C5—C6—H6	119.2	O1—C16—H16C	109.5
C2—C7—C6	119.84 (19)	H16A—C16—H16C	109.5
C2—C7—H7	120.1	H16B—C16—H16C	109.5
C6—C7—H7	120.1	C8—N1—N2	102.97 (15)
N1—C8—N3	113.34 (16)	C9—N2—N1	110.53 (15)
N1—C8—C5	123.47 (16)	C9—N2—H2	124.7
N3—C8—C5	123.19 (16)	N1—N2—H2	124.7
N3—C9—N2	109.15 (17)	C9—N3—C8	104.00 (15)
N3—C9—C10	125.62 (17)	C13—O1—C16	118.50 (18)
N2—C9—C10	125.23 (17)	C2—O2—C1	118.09 (18)
C15—C10—C11	117.84 (18)	H3A—O3—H3B	107.8
			
O2—C2—C3—C4	179.32 (17)	C11—C12—C13—O1	−179.37 (19)
C7—C2—C3—C4	−1.4 (3)	C11—C12—C13—C14	0.1 (3)
C2—C3—C4—C5	0.1 (3)	O1—C13—C14—C15	179.8 (2)
C3—C4—C5—C6	0.5 (3)	C12—C13—C14—C15	0.4 (3)
C3—C4—C5—C8	−179.20 (17)	C11—C10—C15—C14	0.4 (3)
C4—C5—C6—C7	0.1 (3)	C9—C10—C15—C14	−179.59 (18)
C8—C5—C6—C7	179.80 (18)	C13—C14—C15—C10	−0.7 (3)
O2—C2—C7—C6	−178.80 (19)	N3—C8—N1—N2	0.0 (2)
C3—C2—C7—C6	2.0 (3)	C5—C8—N1—N2	179.44 (16)
C5—C6—C7—C2	−1.3 (3)	N3—C9—N2—N1	−0.6 (2)
C6—C5—C8—N1	−173.76 (19)	C10—C9—N2—N1	179.86 (16)
C4—C5—C8—N1	6.0 (3)	C8—N1—N2—C9	0.35 (19)
C6—C5—C8—N3	5.6 (3)	N2—C9—N3—C8	0.6 (2)
C4—C5—C8—N3	−174.69 (17)	C10—C9—N3—C8	−179.88 (17)
N3—C9—C10—C15	175.33 (18)	N1—C8—N3—C9	−0.4 (2)
N2—C9—C10—C15	−5.2 (3)	C5—C8—N3—C9	−179.81 (16)
N3—C9—C10—C11	−4.6 (3)	C12—C13—O1—C16	−178.8 (2)
N2—C9—C10—C11	174.80 (18)	C14—C13—O1—C16	1.8 (3)
C15—C10—C11—C12	0.1 (3)	C7—C2—O2—C1	7.2 (3)
C9—C10—C11—C12	−179.88 (19)	C3—C2—O2—C1	−173.6 (2)
C10—C11—C12—C13	−0.4 (3)		

### Hydrogen-bond geometry (Å, °)

**Table:**

*D*—H···*A*	*D*—H	H···*A*	*D*···*A*	*D*—H···*A*
O3—H3A···N1	0.97	1.96	2.902 (2)	164
N2—H2···O3^i^	0.86	1.90	2.753 (2)	170
O3—H3B···N3^ii^	0.96	1.97	2.885 (2)	159

Symmetry codes: (i) −*x*, −*y*, −*z*+1; (ii) −*x*+1, −*y*, −*z*+1.
